# Supplementary material for: Predicting visual function by interpreting a neuronal wiring diagram
Source: Nature. 2024 Oct 2;634(8032):113–23. doi: 10.1038/s41586-024-07953-5 (PMC11446822; doi:10.1038/s41586-024-07953-5)
Supplement: Supplementary file 6 — CRF and ERF predictions for individual TmY4 and TmY9 cells. Analogous to Supplementary Data 3, but for TmY target types. Shown are the top four monosynaptic pathways, the strongest pathway passing through each of the top ten intermediary types (ranking from Extended Data Fig. 7), and the trisynaptic pathway Tm1–TmY–Dm3–TmY (see the section entitled Prediction of spatial normalization). [file 41586_2024_7953_MOESM6_ESM.zip › DataS4/TmY9qΓèÑ/720575940620403672.pdf]

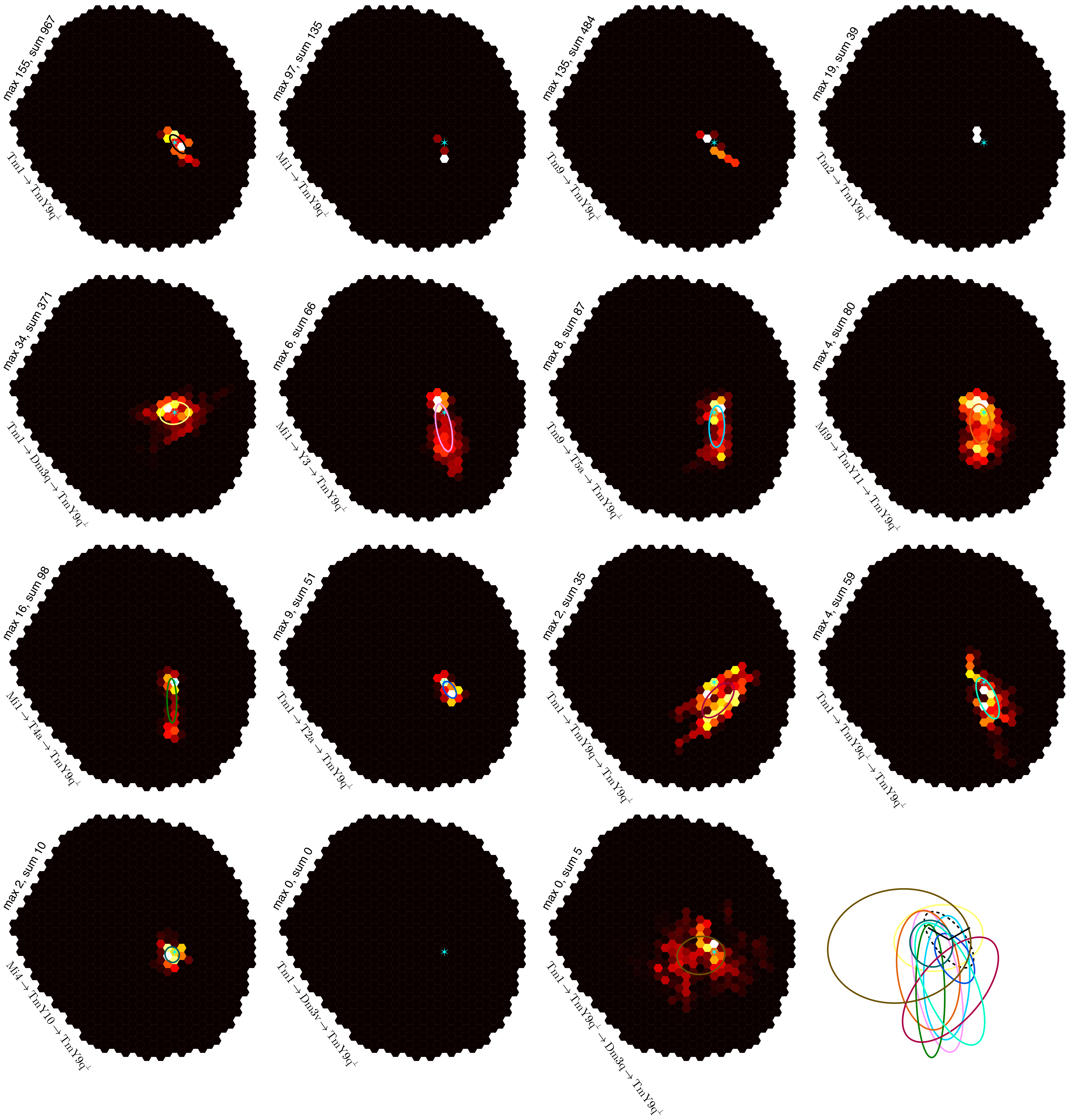
max 155, sum 967
Tm1 → TmY9q⊥
max 97, sum 135
Mi1 → TmY9q⊥
max 135, sum 484
Tm9 → TmY9q⊥
max 19, sum 39
Tm2 → TmY9q⊥
max 34, sum 371
Tm1 → Dm3q → TmY9q⊥
max 6, sum 66
Mi1 → Y3 → TmY9q⊥
max 8, sum 87
Tm9 → T5a → TmY9q⊥
max 4, sum 80
Mi9 → TmY11 → TmY9q⊥
max 16, sum 98
Mi1 → T4a → TmY9q⊥
max 9, sum 51
Tm1 → T2a → TmY9q⊥
max 2, sum 35
Tm1 → TmY9q⊥ → TmY9q⊥
max 4, sum 59
Tm1 → TmY9q⊥ → TmY9q⊥
max 2, sum 10
Mi4 → TmY10 → TmY9q⊥
max 0, sum 0
Tm1 → Dm3v → TmY9q⊥
max 0, sum 5
Tm1 → TmY9q⊥ → Dm3q → TmY9q⊥
